# The effects of cultivation patterns and nitrogen levels on fertility and bacterial community characteristics of surface and subsurface soil

**DOI:** 10.3389/fmicb.2023.1072228

**Published:** 2023-02-16

**Authors:** Chengyu Xu, Yuanjie Chen, Qian Zang, Yulin Li, Jinbiao Zhao, Xuanrui Lu, Min Jiang, Hengyang Zhuang, Lifen Huang

**Affiliations:** ^1^Jiangsu Key Laboratory of Crop Genetics and Physiology/Jiangsu Key Laboratory of Crop Cultivation and Physiology, Agricultural College of Yangzhou University, Yangzhou, China; ^2^Jiangsu Co-Innovation Center for Modern Production Technology of Grain Crops, Yangzhou University, Yangzhou, China

**Keywords:** organic farming, nitrogen level, soil depth, soil nutrient, soil enzyme, soil microorganism

## Abstract

The cropping system affects the physicochemical property and microbial community of paddy soil. Previous research mostly focused on the study of soil 0–20 cm underground. However, there may be difference in the laws of nutrient and microorganism distribution at different depths of arable soil. In surface (0–10 cm) and subsurface (10–20 cm) soil, a comparative analysis including soil nutrients, enzymes, and bacterial diversity was carried out between the organic and conventional cultivation patterns, low and high nitrogen levels. Analysis results suggested that under the organic farming pattern, the contents of total nitrogen (TN), alkali-hydrolyzable nitrogen (AN), available phosphorus (AP), and soil organic matter (SOM) as well as alkaline phosphatase and sucrose activity increased in surface soil, but the SOM concentration and urease activity decreased in subsurface soil. A moderate reduction of nitrogen applied to soil could enhance soil enzyme activity. It was demonstrated by α diversity indices that high nitrogen levels remarkably undermined soil bacterial richness and diversity. Venn diagrams and NMDS analysis manifested great difference in bacterial communities and an apparent clustering tendency under different treatment conditions. Species composition analysis indicated that the total relative abundance of Proteobacteria, Acidobacteria, and Chloroflexi retained stable in paddy soil. LEfSe results revealed that a low nitrogen organic treatment could elevate the relative abundance of Acidobacteria in surface soil and Nitrosomonadaceae in subsurface soil, thereby tremendously optimizing the community structure. Moreover, Spearman’s correlation analysis was also performed, which proved the significant correlation of diversity with enzyme activity and AN concentration. Additionally, redundancy analysis disclosed that the Acidobacteria abundance in surface soil and Proteobacteria abundance in subsurface soil exerted conspicuous influence on environmental factors and the microbial community structure. According to the findings of this study, it was believed that reasonable nitrogen application together with an organic agriculture cultivation system could effectively improve soil fertility in Gaoyou City, Jiangsu Province, China.

## 1. Introduction

Organic food emerges as a new consumption fashion and arouses increasing attention due to its merits of natural ingredients, good quality, high safety, and hygiene. The whole world has witnessed the swift growth of its consumption (Pimentel et al., 2005; Mäder et al., 2007). As one of the competitive organic agricultural specialties in China, organic rice plays an essential part in the organic agricultural product industry. Organic agriculture is instrumental in ecosystem stability. To achieve sustainable development of organic rice, it is urgent to develop complete sets of technical measures appropriate for organic farming. In recent years, China’s rice planting has still been dominated by traditional heavy nitrogen fertilizer application. Although the conventional cultivation mode boosts the short-term yield of rice, the long-term excessive application of single nitrogen fertilizer not only lowers the utilization rate of fertilizer but also brings about severe environmental issues such as soil degradation, groundwater pollution, nitrogen loss, etc. (Ju et al., 2009; Guo et al., 2010). Taking measures to control the amount of nitrogen fertilizer used can save resources, abate pollution, ameliorate soil and water systems, and improve rice quality (Wang H. Y. et al., 2017).

Being critical to material circulating, nutrient transformation, and the mineralization-fixation process of organic carbon, soil microorganisms act as sensitivity indicators of soil quality (Zelles, 1999; Shi et al., 2015). It was established by Gunapala and Scow (1998) that the quantity of soil microorganisms was negatively related to the soil mineral nitrogen content under conventional cultivation, but the two were positively correlated under organic farming. Zhao et al. (2016) noticed that applying pig manure organic–inorganic compound fertilizer while reducing the use of chemical fertilizer could enrich microorganisms related to the carbon-nitrogen cycle, promote nitrification and conversion of organic matter, raise enzyme activity and the amount of microorganisms, and boost plant growth and grain yield. A comparative test conducted by Takahashi et al. (2018) showed that compared with conventional cultivation, organic farming was more capable of diversifying and stabilizing the bacterial population in soil, which greatly suppressed rice diseases. All the above-mentioned results testify the advantage of organic farming. That is, it can recruit effective microorganisms to form a soil environment favorable to crop growth (Wang L. et al., 2017).

The partial factor productivity of nitrogen fertilizer of China is 34 kg·kg^−1^, which is far lower than that of Japan (73 kg·kg^−1^), the Philippines (49 kg·kg^−1^), and Thailand (43 kg·kg^−1^; FAO, 2001). Nitrogen is the main component of crop protein, having vital influence on grain yield. Therefore, clarifying the effect of different nitrogen levels on soil microorganisms can facilitate the development of the optimal crop nutrient management strategy from the perspective of soil ecology. Besides, owing to the high spatial heterogeneity of soil (Imhoff et al., 2000; Critchley et al., 2002) as well as dissimilar soil types, geological features, and climates in different areas, the heterogeneity degree and main driving mechanism of soil vary (Begum et al., 2010). Microorganisms in the arable layer (0–20 cm underground) of rice under organic planting systems were extensively studied previously. However, there are few reports comparing the surface soil (0–10 cm) and subsurface soil (10–20 cm) of the arable layer. More research on vertical heterogeneity of paddy field soil should aid in the knowledge of the change law of soil nutrient uptake and microbial distribution in the arable layer.

An organic farming system of milk vetch-straw-rapeseed cake-San’an bio-organic fertilizer was developed by our research team through a 10-year plot test in Gaoyou, Jiangsu. Given the importance of developing efficient fertilizer application approaches in the future, a comparative test was conducted between conventional and organic farming patterns in this study. Under different management modes of nitrogen levels, the variations of characteristics of bacterial communities (accounting for 70–90% of total microorganisms) in surface and subsurface paddy soil were analyzed *via* high-throughput sequencing. The aim of this study is firstly to discuss the influence of the interaction between cultivation patterns and nitrogen levels on nutrient contents, enzyme activity, and bacterial diversity in paddy soil. Secondly, the change law of soil microbial community characteristics is also clarified by the correlation analysis between soil nutrient indicators and bacteria. Thirdly, the mechanism of microbial communities in paddy soil responding to cultivation patterns is revealed. At last, a reasonable organic agricultural planting system is sought based on soil microorganisms, so as to provide a theoretical foundation for sustainable farming of paddy fields.

## 2. Materials and methods

### 2.1. Plot location and test materials

**Figure 1:**
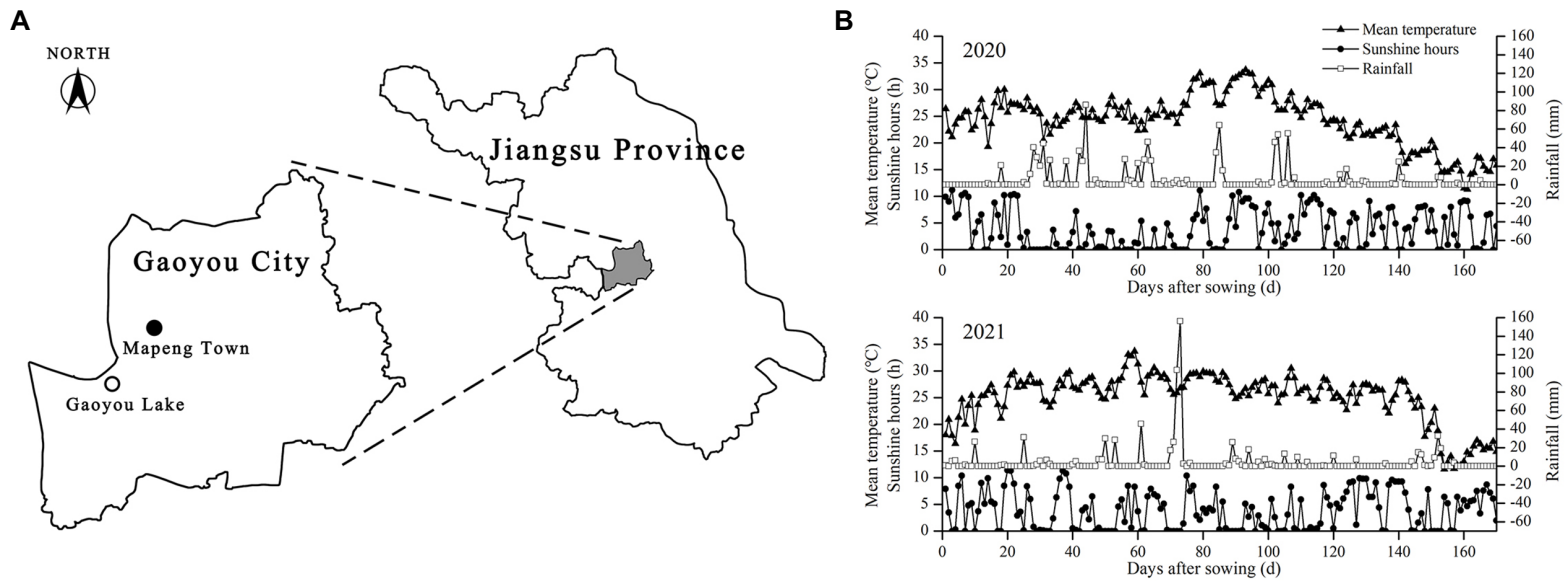
The location of the experimental site **(A)** and meteorological conditions during the whole growth period of rice **(B)**.

The experiment was conducted in the experimental plot of Mapengwan Ecological Agriculture Technology Co., Ltd., Gaoyou City, Jiangsu Province, China (119° 25′ east longitude, 32° 47′ northern latitude) from 2020 to 2021 (Figure 1A). The plot has a northern subtropical monsoon climate, with a mean annual temperature of 16.2°C, annual precipitation of 1341.5 mm, annual sunshine hours of 2100 h, and a frost-free period of 221 days. The plot has long been designated to organic farming research since 2012. The soil property is stable and the texture is clay loam. The soil sample collected in June 2021 contained 27.70 g·kg^−1^ organic matter, 112.24 mg·kg^−1^ alkali-hydrolyzable nitrogen, 7.54 mg·kg^−1^ available phosphorus, 61.16 mg·kg^−1^ available potassium, and 1.32 g·kg^−1^ total nitrogen. The pH of the sample was 8.15. The test rice was of Nanjing 46 brand, with a whole growth period of 165 days. Figure 1B shows the mean daily temperature, daily sunshine hours, and daily precipitation of rice during the period from sowing to harvesting.

### 2.2. Experimental design

The experiment involved farming pattern (*CF* and OF) and nitrogen level (N12 and N18) managements combined into four treatments, including the conventional farming with low nitrogen treatment (CFN12), conventional farming with high nitrogen treatment (CFN18), organic farming with low nitrogen treatment (OFN12), and organic farming with high nitrogen treatment (OFN18), respectively. These treatments were randomly arranged within each zone, and each treatment was repeated 3 times. There were 12 zones, each having an area of 49 m^2^ (7 m × 7 m). Previous crops were wheat, and straw returning was conducted in all the zones. The test rice was sown on 17th May and transplanted by hand on 9th June with row spacing of 30.0 cm × 12.5 cm. Three seedlings were planted per hole.

Conventional farming (CF) was subjected to the high yield cultivation management according to local production rules. 45% compound fertilizer (containing 15%N) was applied as basal fertilizer 1 day before rice transplanting. Taking urea as topdressing nitrogen fertilizer, urea was applied 3 times as the first tillering fertilizer, the second tillering fertilizer and panicle fertilizer at a ratio of 1:2:3.

Organic farming (OF) was performed as per the National Standards for Organic Product Production (GB/T19630.1). The milk vetch-rice planting model was adopted. Milk vetch (containing 0.33% N) was applied as basal fertilizer 2 weeks before rice transplanting *via* once turning-over of soil. Rapeseed cake (containing 4.60% N) and San’an bio-organic fertilizer (containing 4.00% N; Ling et al., 2010) were applied as basal fertilizer 1d before rice transplanting. Subsequently, San’an bio-organic fertilizer was top-dressed as panicle fertilizer in mid-July. The application amount is given in Table 1. N18 is equivalent to 270 kg·hm^−2^ pure nitrogen, which is the local conventional application amount of nitrogen. N12 corresponds to 180 kg·hm^−2^ pure nitrogen, which is a reduced amount of nitrogen for experiment purpose.

**Table 1:** Fertilizer application rates under two nitrogen levels of conventional and organic cultivation (kg·hm^−2^).

Treatment	Basal fertilizer				Topdressing fertilizer	
	Compound fertilizer	Milk vetch	Rapeseed cake	San’an bio-organic fertilizer	Urea	San’an bio-organic fertilizer
CFN12	500	/	/	/	235	/
CFN18	750	/	/	/	352	/
OFN12	/	12,000	1,200	1,200	/	930
OFN18	/	12,000	2,400	1,200	/	1800

The nutrient contents of various fertilizers are: compound fertilizer: 15%N, 15% P_2_O_5_ and 15% K_2_O; Milk vetch: 0.33% N, 0.08% P_2_O_5_ and 0.23% K_2_O; Rapeseed cake: 4.60% N, 0.80% P_2_O_5_, 1.04% K_2_O, 0.80% Ca, 0.48% Mg and various trace elements; San’an bio-organic fertilizer: 4.00% N, 1.87% P_2_O_5_, 2.28% K_2_O, Various organic acids, peptides and rich nutrient elements including 53% organic matter.

### 2.3. Sample collection

The 0–20 cm deep topsoil in each zone was collected by a soil extractor according to the five-point sampling method on October 21, 2021. Four types of soil were obtained, including CFN12, CFN18, OFN12, and OFN18. Each treatment was carried out 3 times. The soil samples were divided into surface soil (0–10 cm) and subsurface soil (10–20 cm). The samples were mixed evenly, from which sundries like rice root residues and stones were removed. A part of the samples (dry soil) were then air-dried and ground prior to filtering through 20-mesh and 100-mesh sieves. Lately, nutrients and enzyme activity of the samples were measured. The other part of the samples (fresh soil) were put into a sealed bag and stored in a refrigerator at −70°C for subsequent analysis of soil microorganisms.

### 2.4. Determination of sample nutrients and enzyme activity

The content of each soil nutrient was measured by the conventional analysis method (Bao, 2000). The alkali-hydrolyzable nitrogen in soil was determined by the alkaline hydrolysis diffusion method. The total nitrogen in soil was detected by the H_2_SO_4_-mixed accelerator distillation method. Soil organic matter was measured by the K_2_Cr_2_O_7_-H_2_SO_4_ external heating approach. The available phosphorus in soil was detected using NaHCO₃ extraction spectrophotometry. The available potassium in soil was detected by CH_3_COONH_4_ extraction flame spectrophotometry. The soil pH was measured manually by the COMBI 5000 gauge in accordance with operation manual.

The enzyme activity of soil was detected using the method proposed by Guan (1986). The 3,5-dinitrosalicylic acid colorimetric method, indophenol blue colorimetry method, and disodium phenyl phosphate colorimetry method were used to measure the activity of soil sucrose, urease, and alkaline phosphatase, respectively.

### 2.5. DNA extraction, PCR amplification, and high-throughput sequencing

0.5 g soil was taken from each sample and the microorganism DNA was extracted from the soil using the Omega soil DNA kit. The quantity and quality of the DNA extracted were assessed by ultraviolet spectrophotometer (NanoDrop, Thermo Scientific, NC2000), while the DNA integrity was determined by 1.2% agarose gel electrophoresis. The target DNA sequence of bacteria for amplification was the 16S_V3V4 region. PCR amplification of 16S rDNA was carried out with 338F (5′-ACTCCTACGGGAGGCAGCA-3′) as the forward primer and 806R (5′-GGACTACHVGGGTWTCTAAT-3′) as the reverse primer. The amplification system (25 μL) consisted of 5 × reaction buffer 5 μL, 5 × GC buffer 5 μL, dNTP (2.5 mM) 2 μL, forward primer (10 uM) 1 μL, reverse primer (10 uM) 1 μL, DNA template 2 μL, ddH2O 8.75 μL, and Q5 DNA polymerase 0.25 μL. The amplification conditions were pre-denaturation at 98°C for 2 min, denaturation at 98°C for 15 s, annealing at 55°C for 30 s, extension at 72°C for 30 s, final extension at 72°C for 5 min, and heat preservation at 10°C. The process was repeated for 25–30 cycles. After amplification, gel electrophoresis was conducted and 2% agarose was prepared to detect the PCR amplification products. Following QIIME2 dada2 analysis process, Illumina MiSeq sequencing was performed for quality control, denoise, mergence, and chimera removal. Shanghai Personalbio Technology Co., Ltd. was entrusted to conduct high-throughput sequencing of soil.

### 2.6. Data analysis

Data were sorted and analyzed by Microsoft Excel 2019 and SPSS 23.0 software and mapped by Origin 8.5. Soil nutrients and enzyme activity data of various treatment modes were analyzed by the univariate method. Differences in data among groups were compared by LSD. Microbial community characteristics were mapped on the Genescloud platform. According to the distribution of amplicon sequence variants or operational taxonomic units (ASV/OTU) in different treatment modes, Alpha diversity of each treatment was calculated, and a rarefaction curve was drawn to assess whether the sequencing depth was reasonable. The Venn diagram was used to count the number of exclusive and shared ASV/OTU of different treatment methods based on ASV/OTU richness. Species composition analysis was made through assessing the feature table with singletons removed, and the phylum-level diversity was shown by a histogram. In non-metric multidimensional scaling (NMDS) analysis, the Bray–Curtis distance matrix was used for dimension reduction and decomposition, and differences in bacterial community composition were described with a two-dimension distribution figure. In LEfSe analysis, with the linear discriminant analysis (LDA) threshold set at 3.5, Kruskal-Wallis test was conducted to analyze simultaneously the difference among varied groups and identify notable biomarkers in each treatment. The correlation of α diversity indices with nutrients and enzyme activity was investigated by Spearman’s method. The relevance of dominant phyla with nutrients and enzyme activity was examined by redundancy method.

## 3. Results

### 3.1. The influence of organic and conventional farming modes and two nitrogen levels on soil nutrient and enzyme activity

#### 3.1.1. Soil nutrients

As is shown in Table 2, organic farming surface soil contained more TN, AN, AP, and SOM than conventional farming surface soil. The content of the above-mentioned four nutrients in OFN12 type soil was higher than that in CFN12 type soil by 8.46, 9.23, 8.99, and 4.55%, respectively. The content of the four nutrients in OFN18 type soil was higher than that in CFN18 type soil by 7.39, 15.21, 12.63, and 2.84%, respectively. High nitrogen application raised the concentration of AN. The content of AN in CFN18 type soil was 4.06% higher than that in CFN12 type soil, and the content of AN in OFN18 type soil was 9.76% higher than that in OFN12 type soil. As for subsurface soil, conventional farming soil contained more SOM than organic farming soil. The content of SOM in CFN12 type soil was 6.66% higher than that in OFN12 type soil, and the content of SOM in CFN18 type soil was 11.18% higher than that in OFN18 type soil. The AP and AK content in conventional farming soil was significantly lower than those in organic farming soil. Their concentrations in CFN12 type soil were reduced by 15.27 and 5.29%, respectively, compared with those in OFN12 type soil. Their concentrations in CFN18 type soil were reduced by 12.92 and 7.04%, respectively, compared with those in OFN18 type soil. Generally, the surface soil contained more nutrients than subsurface soil, while the pH of surface soil was lower than that of subsurface soil. Whether it was surface soil or subsurface soil, the pH of organic farming soil was remarkably lower than that of conventional farming soil under the same nitrogen level condition. Under the same farming pattern, the pH of soil treated by high nitrogen was lower than that treated by low nitrogen, and the difference was notable in the pH of subsurface soil.

**Table 2:** Effects of two nitrogen levels under organic and conventional cultivation on soil nutrients.

Layer(cm)	Treatment	ω(TN)/ (g·kg^−1^)	ω(AN)/(mg·kg^−1^)	ω(AP)/(mg·kg^−1^)	ω(AK)/(mg·kg^−1^)	ω(SOM)/(g·kg^−1^)	pH
0–10	CFN12	2.01 ± 0.03 b	155.97 ± 2.21 c	12.12 ± 0.23 b	110.5 ± 1.5 a	39.76 ± 0.02 bc	8.33 ± 0.02 a
	CFN18	2.03 ± 0.03 b	162.30 ± 1.25 c	12.11 ± 0.04 b	105.5 ± 0.5 bc	39.43 ± 0.01 c	8.32 ± 0.01 ab
	OFN12	2.18 ± 0.03 a	170.36 ± 1.01 b	13.21 ± 0.04 a	103.5 ± 0.5 c	41.57 ± 0.09 a	8.25 ± 0.02 bc
	OFN18	2.18 ± 0.01 a	186.98 ± 0.56 a	13.64 ± 0.08 a	109.5 ± 0.5 ab	40.55 ± 0.35 ab	8.24 ± 0.02 c
10–20	CFN12	1.49 ± 0.01 ab	106.93 ± 2.00 a	8.38 ± 0.21 b	98.5 ± 1.5 c	26.42 ± 0.19 b	8.73 ± 0.01 a
	CFN18	1.52 ± 0.00 a	110.00 ± 1.07 a	8.09 ± 0.18 b	99.0 ± 1.0 bc	27.05 ± 0.01 a	8.64 ± 0.01 b
	OFN12	1.42 ± 0.01 ab	101.33 ± 3.49 a	9.89 ± 0.07 a	104.0 ± 0.0 ab	24.77 ± 0.01 c	8.65 ± 0.02 b
	OFN18	1.41 ± 0.04 b	102.03 ± 0.09 a	9.29 ± 0.09 a	106.5 ± 0.5 a	24.33 ± 0.03 c	8.57 ± 0.02 c

Data are presented by mean value ± SD. Different lower case letters in the same column indicate significant differences among the four treatments at *p* < 0.05. TN, total nitrogen; AN, alkali-hydrolyzable nitrogen; AP, available phosphorus; AK, available potassium; SOM, soil organic matter. The same below.

#### 3.1.2. Soil enzyme activity

**Figure 2:**
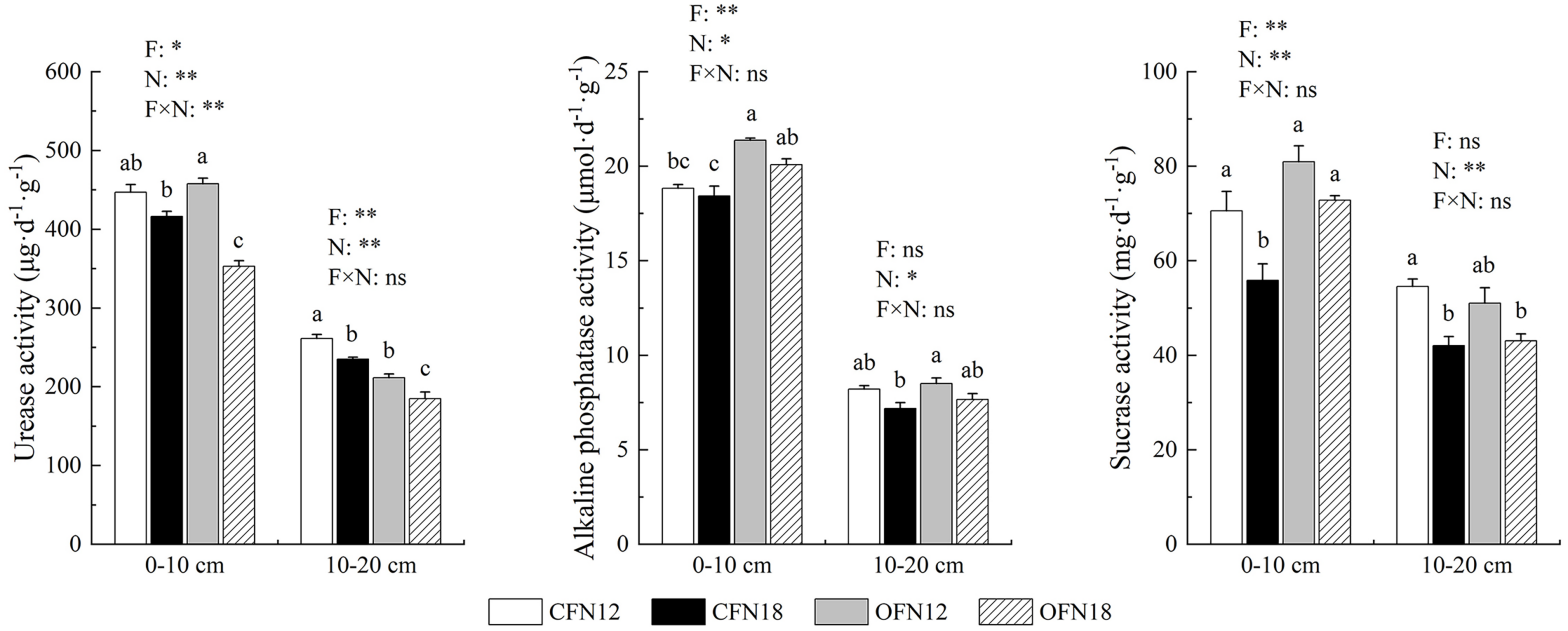
Effects of two nitrogen levels under organic and conventional cultivation on soil enzyme activities. F, Cultivation Pattern; N, Nitrogen Level; F × N, interaction between F and N. *p < 0.05, **p < 0.01, ns *p* > 0.05. Different lower case letters indicate significant differences among the four treatments at *p* < 0.05.

As shown in Figure 2, the activity of 3 enzymes in surface soil was all apparently higher than that in subsurface soil. The farming methods and nitrogen levels had great impact on soil enzyme activity, but the interaction between the two was slight in general. Three enzymes in both surface and subsurface soil were found more active at low nitrogen levels than at high nitrogen levels. At the same nitrogen level, the activity of alkaline phosphatase and sucrase in organic farming surface soil was tremendously higher than that in conventional farming surface soil. Compared with that in conventional farming soil, the activity of alkaline phosphatase in organic farming soil was promoted by 13.44 and 8.96% at low and high nitrogen levels, respectively, and the activity of sucrase in organic farming soil was improved by 14.67 and 30.31% at low and high nitrogen levels, respectively. Nevertheless, organic farming greatly reduced the activity of urease in subsurface soil. The activity of urease in organic farming subsurface soil was 19.14 and 21.37% lower than that in conventional farming subsurface soil at low and high nitrogen levels, respectively.

### 3.2. The influence of organic and conventional farming modes and two nitrogen levels on characteristics of bacterial communities in soil

#### 3.2.1. Alpha diversity index

**Figure 3:**
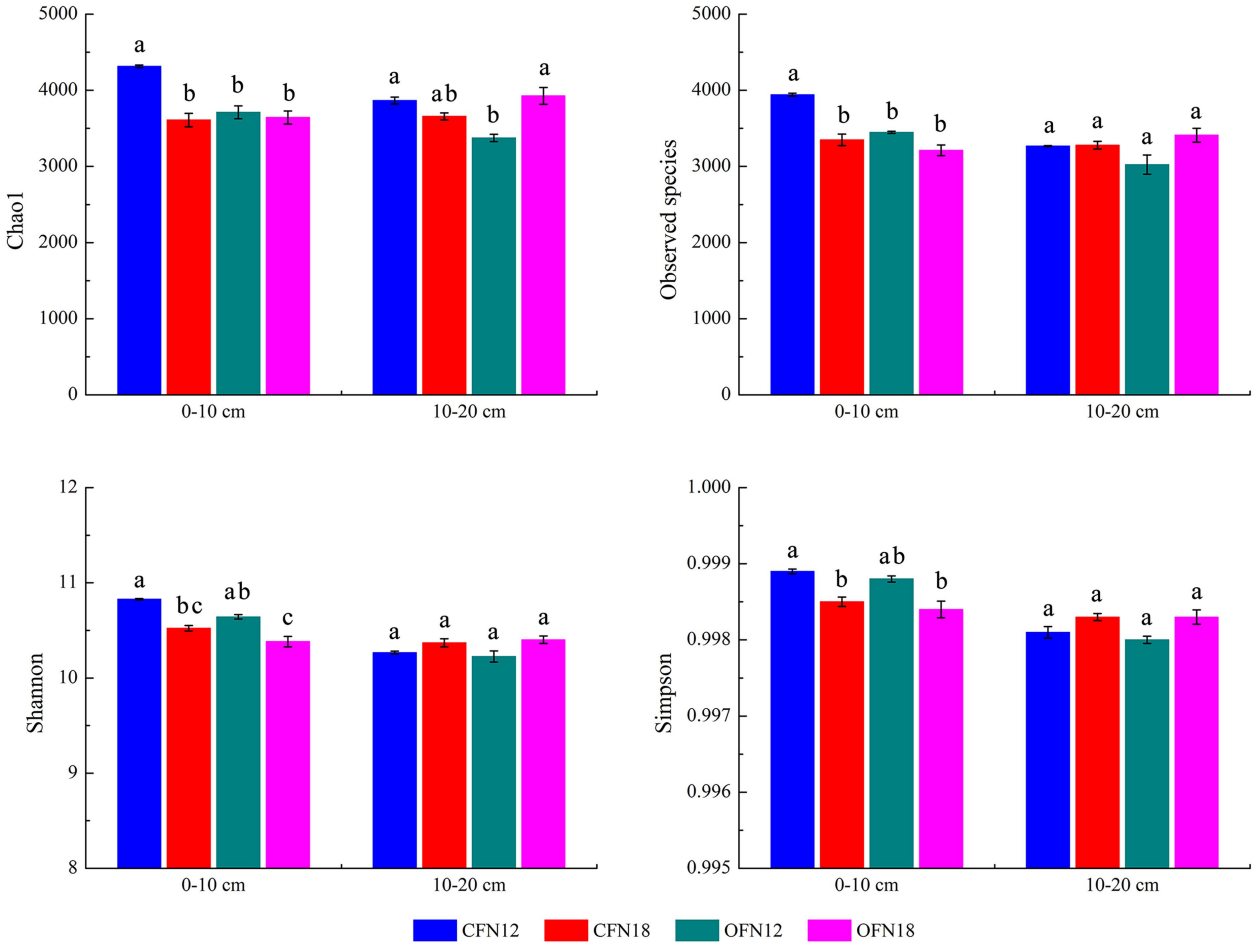
Alpha diversity of bacteria under two nitrogen levels of conventional and organic cultivation. Different lower case letters indicate significant differences among the four treatments at *p* < 0.05.

Bacterial richness was represented by Chao1 and Observed species indices, while bacterial diversity was denoted by Shannon and Simpson indices in this paper. Figure 3 shows the indices. For surface soil, the Chao1 and Observed species indices of the CFN12 group were significantly higher than those of the other three groups. Compared with low nitrogen application, high nitrogen application reduced bacterial richness and diversity. Chao1, Observed species, Shannon, and Simpson indices of CFN18 type surface soil were remarkably lower than those of CFN12 type surface soil by 16.34, 15.05, 2.82, and 0.04%, respectively. Chao1, Observed species, Shannon, and Simpson indices of OFN18 type surface soil were lower than those of OFN12 type surface soil by 1.82, 6.83, 2.44, and 0.04%, respectively. For subsurface soil, Chao1 index of the OFN12 group was lower than that of other three groups. Under both organic and conventional cultivation patterns, Observed species, Shannon, and Simpson indices at high nitrogen levels were slightly higher than those at low nitrogen levels.

#### 3.2.2. Bacterial community difference

**Figure 4:**
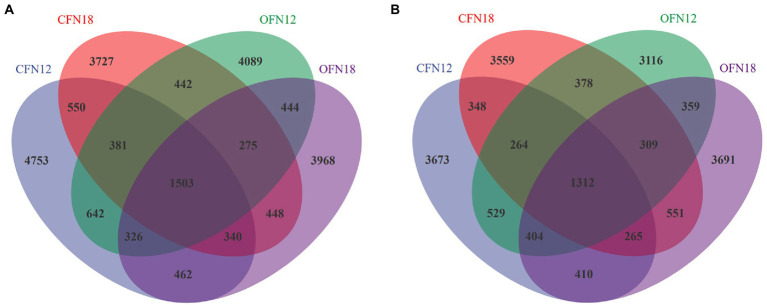
OTUs Venn Diagram of soil bacteria. **(A)** 0–10 cm; **(B)** 10–20 cm.

To investigate the exclusive and shared species of different treatment groups, the Venn diagram (Figure 4) was plotted for community analysis. In general, the farming methods and nitrogen levels affected notably the species in soil. There were obviously more species in surface soil than in subsurface soil. As shown in Figure 4A, the number of OTU in surface soil of the CFN12, CFN18, OFN12, and OFN18 groups was 8,957, 7,666, 8,102, and 7,766, respectively. The number of exclusive OTU in surface soil of the above four groups was 4,753, 3,727, 4,089, and 3,968, respectively. There were 1,503 shared OTU in total. The bacterial richness and number of exclusive species in CFN12 type surface soil were especially higher than those in other three types of surface soil. As is shown in Figure 4B, there were 7,205, 6,986, 6,671, and 7,301 OTU in subsurface soil of the CFN12, CFN18, OFN12, and OFN18 groups, respectively. There were 3,673, 3,559, 3,116, and 3,691 exclusive OTU in subsurface soil of the above four groups, respectively. The share OTU of the four groups was 1,312. The treatment of OFN12 reduced the bacterial richness and the number of exclusive species in subsurface soil.

#### 3.2.3. NMDS analysis

**Figure 5:**
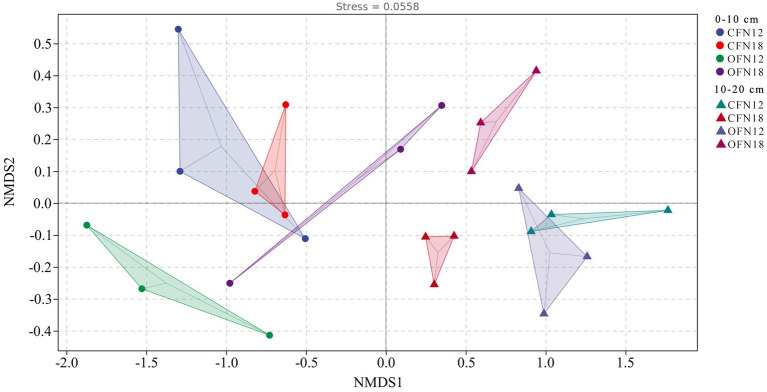
Non-metric multidimensional scaling analysis score plots of bacteria based on the Bray–Curtis distance.

NMDS analysis can simplify the data structure, describe the distribution of samples at a given distance metric, and represent clearly the difference in the bacterial community structure among varied treatment groups. As shown in Figure 5, dots of different colors denote different treatment groups, and a closer distance between two dots indicates more similarities in the bacterial community between two groups. The stress of NMDS analysis is 0.0558 (<0.2), proving that NMDS fitting can accurately measure the bacterial community structure of different treatment groups. For surface soil, the bacterial community structures of CFN18 and CFN12 groups were quite similar, while the bacterial community structures of OFN18 and OFN12 groups were greatly dissimilar. For subsurface soil, the bacterial community of the CFN12 group notably resembled that of the OFN12 group, while the bacterial community of the CFN18 group greatly differed from that of the OFN18 group. Significant differences were found in the bacterial community between surface and subsurface soil. Soil has high heterogeneity and the bacterial community varies in soil at different depths.

#### 3.2.4. Species composition analysis

**Figure 6:**
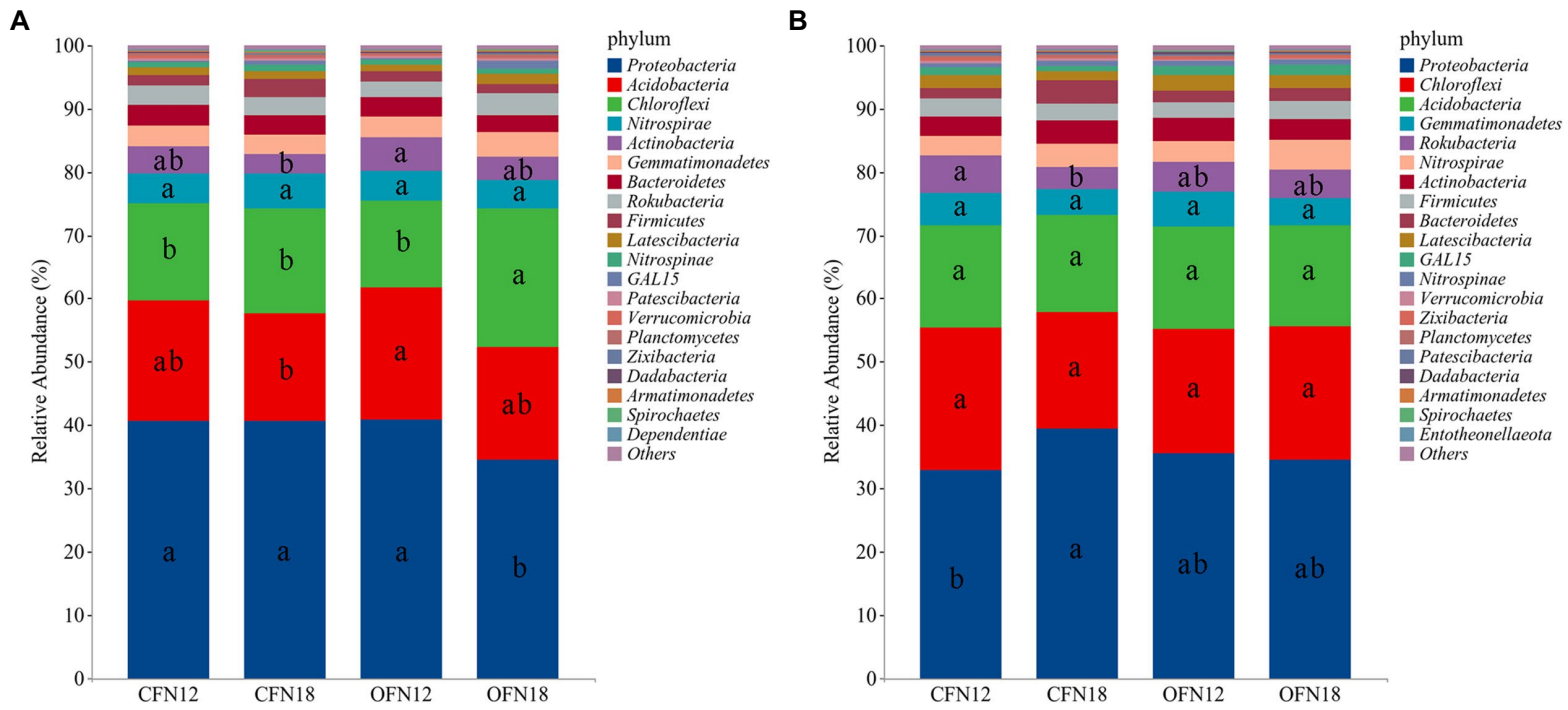
Relative Abundance of bacteria on phylum level. **(A)** 0–10 cm; **(B)** 10–20 cm. Different lower case letters indicate significant differences among the four treatments at p < 0.05.

The relative abundance of phyla in each treatment group was analyzed after high-throughput sequencing of the bacterial community. The top 5 dominant phyla were compared among different groups. Figure 6 shows top 20 species with the highest relative abundance, and the relative abundance of remaining species was combined and classified as Others. In surface soil (Figure 6A), the top 5 phyla with the highest relative abundance were Proteobacteria, Acidobacteria, Chloroflexi, Nitrospirae, and Actinobacteria. Proteobacteria (34.46–40.93%), Acidobacteria (17.07–20.72%), and Chloroflexi (13.77–21.99%) phyla predominated in surface soil, accounting for 75% of the total phyla. Among the four different treatment groups, the relative abundance of Proteobacteria was the lowest and the relative abundance of Chloroflexi was the highest in the OFN18 group. Compared with the low nitrogen treatment, high nitrogen application reduced the relative abundance of Proteobacteria, Acidobacteria, and Actinobacteria. Their relative abundance in CFN18 type soil was lower than that in CFN12 type soil by 0.20, 9.89, and 26.80%, respectively. Their relative abundance in OFN18 type soil was lower than that in OFN12 type soil by 15.81, 14.23, and 31.39%, respectively. However, the relative abundance of Chloroflexi in CFN18 and OFN18 groups was higher than that in CFN12 and OFN12 groups by 7.30 and 59.74%, respectively. High nitrogen application abated the total ratio of dominant phyla but enlarged the total ratio of rare phyla.

In subsurface soil (Figure 6B), the top 5 phyla with the highest relative abundance were Proteobacteria, Chloroflexi, Acidobacteria, Gemmatimonadetes, and Rokubacteria. The dominant phyla were Proteobacteria (32.77–39.31%), Chloroflexi (18.52–22.66%), and Acidobacteria (15.37–16.15%), which occupied 70% of the total phyla. The relative abundance of Proteobacteria and Rokubacteria in CFN18 type soil was 19.98% higher and 40.84% lower than that of CFN12 type soil, respectively, and the differences were significant. The relative abundance of Chloroflexi, Acidobacteria, and Gemmatimonadetes maintained stable and showed little difference among four groups. On the whole, the ratio of dominant phyla in surface soil was larger than that in subsurface soil, while there were more rare phyla in subsurface soil than in surface soil.

#### 3.2.5. LEfSe analysis

**Figure 7:**
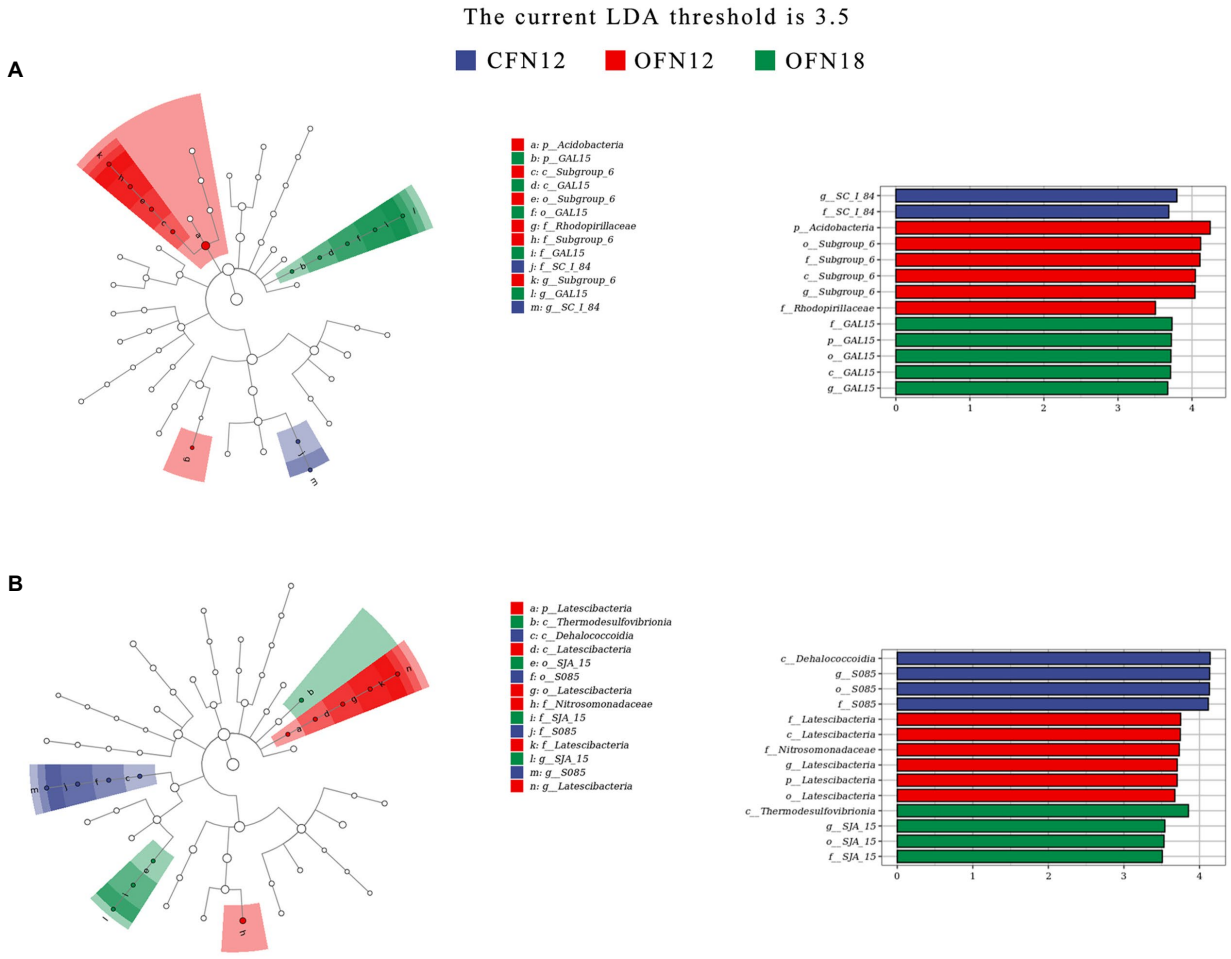
LEfSe analysis of bacterial community. **(A)** 0–10 cm; **(B)** 10–20 cm. p, phylum; c, class; o, order; f, family; g, genus.

Biomarkers that were tremendously different among four treatment groups were identified by LEfSe analysis (species with abundance lower than 0.0015 were removed). Results were concluded in a cladogram and a histogram of the LDA scores. The cladogram represents the phylum to genus from the innermost ring to the outermost ring. The diameter of small circles is proportional to the relative abundance of the species, and the biomarkers with significant difference are colored the same as the group. The length of a row in the histogram represents the LDA score, namely, the influence of the biomarker. It was found that in surface soil (Figure 7A), f_SC_I_84 was notably enriched in the CFN12 group, p_Acidobacteria and f_Rhodospirillaceae were remarkably enriched in the OFN12 group, and p_GAL15 was significantly enriched in the OFN18 group. No biomarkers were observed in the CFN18 group. According to the LDA histogram, when the LDA value was greater than 3.5, all the four groups had 13 biomarkers in total and there were more biomarkers in OF groups than in *CF* groups. The most influenced species in CFN12, OFN12, and OFN18 type soil were g_SC_I_84, p_Acidobacteria, and f_GAL15, respectively.

In subsurface soil (Figure 7B), the enriched species in CFN12, OFN12, and OFN18 groups were c_Dehalococcoidia, p_Latescibacteria and f_Nitrosomonadaceae, c_Thermodesulfovibrionia and o_SJA_15, respectively. No biomarkers were found in the CFN18 group. There were totally 14 biomarkers in four groups when the LDA value was larger than 3.5, as is shown in the LDA histogram. The OFN12 group had the most biomarkers. c_Dehalococcoidia in the CFN12 group, f_Latescibacteria in the OFN12 group, and c_Thermodesulfovibrionia in the OFN18 group were subjected to the greatest impact.

It is noteworthy that among all biomarkers, p_Acidobacteria with the highest relative richness in surface soil and f_Nitrosomonadaceae with the highest relative abundance in subsurface soil were both present in the OFN12 group.

### 3.3. Relevance between soil nutrients and microbial communities

#### 3.3.1. Correlation between environmental factors and diversity indices

**Figure 8:**
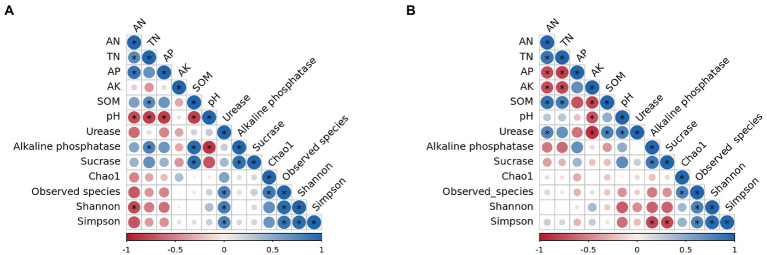
Correlation analysis of soil nutrients, enzyme activities and diversity index. **(A)** 0–10 cm; **(B)** 10–20 cm. *p < 0.01.

The relationship between soil environmental factors and bacterial α diversity was analyzed by the Spearman’s algorithm (Figure 8). The results suggested that in surface soil (Figure 8A), four α diversity indices were significantly related with each other. Observed species, Shannon, and Simpson indices were positively correlated to Urease, while Shannon was negatively correlated to AN. What’s more, a negative connection was observed between pH values and AN, TN, AP, SOM, and Alkaline phosphatase. SOM was positively associated with TN, Alkaline phosphatase, and Sucrase. Besides, AN was positively correlated to TN and AP, and Alkaline phosphatase was positively connected to TN and Sucrase. All the correlations were notable.

In subsurface soil (Figure 8B), a significant association was also observed among four α diversity indices. Simpson was negatively related to Alkaline phosphatase and Sucrase. AK was positively correlated with AN, TN, SOM, pH, and Urease. SOM was positively connected to AN, TN, and Urease. AN was positively correlated with TN and Urease. AP was negatively related to AN and TN. Moreover, a positive connection was found between Alkaline phosphatase and Sucrase as well as between pH values and Urease. All the correlations were significant.

#### 3.3.2. Redundancy analysis of environmental factors and dominant phyla

**Figure 9:**
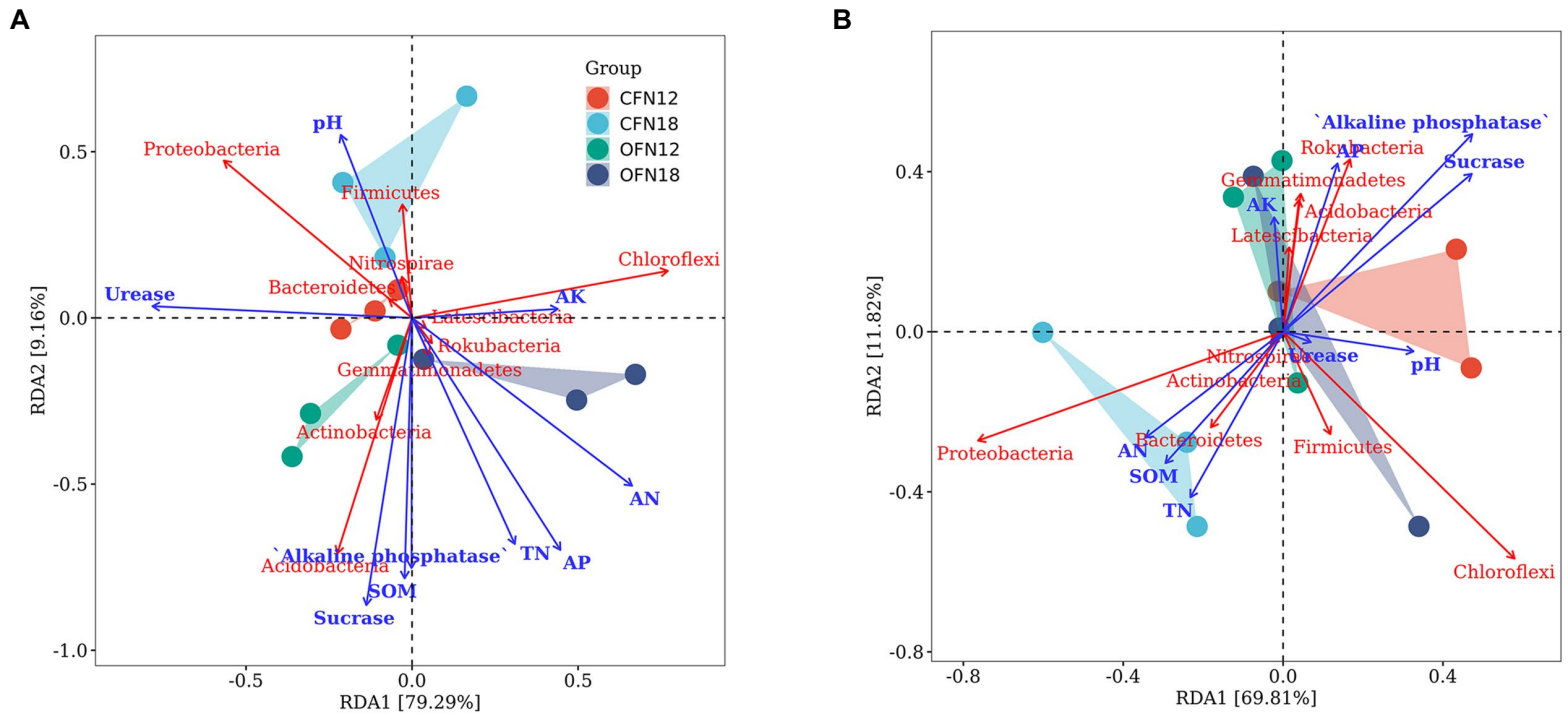
Redundancy analysis of soil nutrients, enzyme activities and abundant phyla. **(A)** 0–10 cm; **(B)** 10–20 cm.

The relationship of top 10 phyla with environmental factors was investigated by redundancy analysis. The results are summarized in Figure 9. In surface soil (Figure 9A), RDA1 and RDA2 accounted for 79.29 and 9.16% variations of the bacterial community, respectively. AK (r2 = 0.18, *p* = 0.39) had small influence on community composition. Acidobacteria was positively associated with AN, TN, AP, SOM, Alkaline phosphatase, and Sucrase (AN: r2 = 0.67, *p* = 0.01; TN: r2 = 0.55, *p* = 0.02; AP: r2 = 0.67, *p* = 0.00; SOM: r2 = 0.60, p = 0.01; Alkaline phosphatase: r2 = 0.55, *p* = 0.03; Sucrase: r2 = 0.75, p = 0.00), while Proteobacteria was negatively related to these factors. There was slight correlation between Chloroflexi and most environmental factors.

In subsurface soil (Figure 9B), RDA1 and RDA2 explained 69.81 and 11.82% variations of the bacterial community, respectively. Alkaline phosphatase (r2 = 0.45, *p* = 0.07) might impact community composition, but all the other environmental factors had little effect on community composition, with r2 ranging between 0.01 ~ 0.37 and p between 0.13 ~ 0.98. Proteobacteria was positively correlated to AN, TN and SOM, but negatively correlated to other environmental factors. Chloroflexi was not related to a majority of the environmental factors.

## 4. Discussion

### 4.1. Effects of cultivation patterns and nitrogen levels on nutrients in surface and subsurface soil

Organic fertilizer can ameliorate physicochemical properties of soil, raise the amount of SOM and renovate SOM, lessen nutrient loss, and enhance fertilizer utilization efficiency (Rahman et al., 2011; Hou et al., 2016; Du et al., 2020). In this study, the content of TN, AN, AP, and SOM in surface soil and the content of AP and AK in subsurface soil were notably elevated under the organic farming pattern. This finding is consistent with the conclusion of previous studies. The content of nutrients in soil at different depths increased in varying degrees. Dislike TN, AN can represent the capacity of soil to supply nitrogen available to rice in a short term. In the organic farming system, planting milk vetch increased the concentration of available (potentially mineralizable) nitrogen of the paddy field ecosystem. Such legume fertilizer released nitrogen in synchrony with the nitrogen uptake of plants, and meanwhile reduced nitrogen loss through leaching and volatilization (Askegaard and Eriksen, 2007; Suja et al., 2012). Results of this study revealed that AN content in surface soil with high nitrogen applied was higher than that with low nitrogen applied, indicating that an increased nitrogen application amount directly led to ascending AN content. Nevertheless, the content of SOM in subsurface soil did not rise under the organic farming pattern. The reason might be that under the conventional farming mode, soil microorganisms decomposed subsurface SOM at a slow rate, but the microorganisms tended to be more active and disintegrated the original SOM in soil under the organic farming mode. It requires some time to transform organic matter into SOM. As a result, the SOM content in subsurface soil was lower under the organic farming pattern than under the conventional farming mode. Soil nutrient stratification can be observed on the section of the upper layer of soil undergoing long-term farming, and generally, the nutrient content descends as the depth increases (Gwenzi et al., 2009). In our experiment, nutrient stratification of paddy soil was also noticed because of fertilizer application, and there were more nutrients in surface soil than in subsurface soil. Previous researchers have already confirmed that application of a large amount of nitrogen fertilizer over a long period can bring about soil acidification (Bowman et al., 2008). This conclusion corresponded to a slight decrease in pH under high nitrogen treatment. Meanwhile, the pH of surface soil was greatly lower than that of subsurface soil owing to nitrogen stratification. Most notably, the pH of organic farming soil was lower than that of conventional farming soil. Two reasons might be accountable. One is that organic fertilizer used induces nitrification and the release of H^+^ (Xun et al., 2016), and the other is organic acid accumulation in organic fertilizer (Liang et al., 2012). It is thus evident that organic farming has a latent capacity to reduce the pH level of alkaline soil in Gaoyou, Jiangsu Province.

Niemi et al. (2005) claimed that root biomass and enzyme activity declined as soil depth increased. It was also found in our study that enzyme activity in surface soil was more intense than in subsurface soil, which might be attributed to an increased bulk density and decreased metabolic diversity of soil (Li et al., 2002; Griffiths et al., 2003). Remarkable changes in enzyme activity were also related to the amount of nitrogen applied (Corrales et al., 2017). In this paper, nitrogen reduction was suggested to boost enzyme activity. It was because low nitrogen application limited the supply of nitrogen needed by soil microorganisms (Mooshammer et al., 2014), which thereby had to secret more enzymes to acquire more nitrogen (Olander and Vitousek, 2000). It has been proven by a large number of studies that organic fertilizer promotes enzyme activity (Ye et al., 2014; Wang N. et al., 2020; Li et al., 2022). Results of this study also indicated that organic farming greatly activated alkaline phosphatase and sucrase in surface soil. The reason was that organic fertilizer increased the SOM content and stabilized the aggregates in surface soil, provided soil microorganisms with adequate carbon sources and a good environment, and enriched the substrates needed by soil enzymes. However, urease activity in subsurface soil was reduced under the organic farming mode. The proximate cause for why urease in organic farming soil was less active than that in conventional farming soil might be that urease could catalyze the hydrolysis of urea, and urea applied as topdressing in convention farming increased urease activity notably.

### 4.2. The impact of cultivation patterns and nitrogen levels on microorganisms in surface and subsurface soil

The present study results showed that bacterial diversity indices were subject to nitrogen levels in a short term. Campbell et al. (2010) noticed a decrease in bacterial diversity of Arctic tundra soil with an increased nitrogen application amount. Nevertheless, Fierer et al. (2012) reported no association between the nitrogen application amount and bacterial diversity of grassland and farmland soil in the United States. It is clear that effects of nitrogen levels on bacterial communities vary in different regions. The study of Liu et al. (2011) on paddy soil in southeastern China suggested that applying an appropriate amount of nitrogen could increase microbial biomass and respiration activity of paddy soil, but excessive nitrogen would impede soil bacterial diversity. The findings of the present study proved that low nitrogen levels could improve bacterial richness and diversity of surface soil, especially under the conventional farming pattern (Figures 3, 4). There are long-standing issues such as excessive application of industrial nitrogen fertilizer and a lack of awareness of soil conservation in rice cropping regions in Jiangsu Province. An excessive supplement of external nitrogen fertilizer would result in extremely low soil C/N. Without sufficient carbon sources, soil microorganism growth is restricted and soil microbial biomass is thus greatly reduced (Fontaine et al., 2003). Therefore, a proper reduction of nitrogen may increase microbial diversity of surface soil. A previous study established that microbial communities had stable and strong adaptability to the increasing depth of paddy soil (Yu et al., 2020). In this experiment, Observed species, Shannon, and Simpson indices of subsurface soil under high nitrogen levels were just slightly larger than those under low nitrogen levels, and the difference was not significant. It indicated that a proper reduction of nitrogen could improve nitrogen fertilizer use efficiency and reduce nitrogen loss resulted from leaching without damnifying microbial diversity in subsurface soil.

Both the Venn diagram and nonmetric multidimensional scaling analysis results suggested that microbial communities varied greatly and tended to cluster under different treatment conditions (Figures 4, 5). It was indicated in a study that the difference in soil microbial communities was ascribed to the mutual selection of plants and environmental factors (Mendes et al., 2014). Different from that in a natural ecosystem, the microbial community in the paddy field ecosystem under human control is more susceptible to environmental changes (Xu et al., 2019). Environmental factors of this test included farming patterns, nitrogen levels, and soil depth. These factors increased the exclusive species of different treatment groups and remarkably affected the microbial community composition of paddy soil. Bacteria in subsurface soil showed an obvious tendency to cluster. In contrast, bacteria in surface soil were distributed in a more scattered manner (Figure 5). A possible reason was the influence of bacterial community competition, artificial disturbance and climate change.

The cultivation patterns had little influence on bacterial diversity but great impact on the bacterial community structure. Proteobacteria, Acidobacteria, and Chloroflexi were the top dominant phyla in paddy soil. This finding conformed to that of previous research (Huang et al., 2019; Li et al., 2019). Significantly decreased Proteobacteria and remarkably increased Chloroflexi in surface soil of the OFN18 group (Figure 6) were attributed to the competition of dominant phyla. Though there were changes in the proportion of dominant phyla among different treatment groups, the total relative abundance of the above 3 dominant phyla remained unchanged. The total relative abundance of surface soil was 5% higher than that of subsurface soil, which was probably because some aerobic bacteria in the dominant phyla proliferated rapidly in surface soil with good permeability and high nutrient and O_2_ content. Ramirez et al. (2012) carried out a series of tests to study the effect of different application amounts of nitrogen, and they found that high nitrogen levels reduced the relative abundance of Acidobacteria. Their finding was in agreement with the result of this study that Acidobacteria was tremendously enriched in surface soil of the OFN12 group (Figures 6, 7). However, Acidobacteria did not become a biomarker in CFN12 treatment. The reason might be that some species in Acidobacteria with genes encoding cellulase and hemicellulos (Kanokratana et al., 2011) participated in degrading a large number of milk vetch and rapeseed meal residues under the organic farming pattern. Oxidation of ammonium to nitrites by ammonia-oxidizing bacteria (AOB) is an essential part of nitrification (Chen et al., 2010). The amoA gene involved in catalytic nitrification is closely related to Nitrosospira and Nitrosomonas in AOB (Hussain et al., 2011). The results of this paper showed that Nitrosomonadaceae, to which Nitrosomonas belongs, was the biomarker of subsurface soil under the OFN12 treatment (Figure 7). Previous researchers have established that the AOB community in the rice rhizosphere was highly responsive to soil depth and nitrogen fertilizer application (Wang et al., 2009; Hussain et al., 2011). This study implied that under the organic farming pattern, low nitrogen treatment could increase the quantity of Nitrosomonas in subsurface soil, thereby improving nitrification efficiency and nitrogen fixation activity. To conclude, organic farming has the merit of optimizing the community structure.

### 4.3. Connection between soil nutrients and microbial community

The Spearman’s correlation test revealed a correlation between diversity indices and enzyme activity. In surface soil, Shannon was significantly positively related to urease but negatively related to AN. It was inferred that nitrogen application easily led to changes in bacterial diversity. As the mount of nitrogen fertilizer applied increased, AN lifted greatly, resulting in unbalanced C/N. Low carbon induced declined bacterial diversity in surface soil. Under high nitrogen levels, urease maintained low activity and thus prevented severe imbalance of C/N (Mooshammer et al., 2014; Chen et al., 2018). In subsurface soil, Simpson was significantly negatively correlated with alkaline phosphatase. The reason might be that the amount of nitrogen flowing from surface soil into subsurface soil was moderate, which unexpectedly increased bacterial diversity. Subsurface soil of Gaoyou city is prone to phosphorus deficiency, and applying nitrogen fertilizer (compound fertilizer and San’an bio-fertilizer) added phosphorus into soil and alleviated phosphorus shortage, which therefore reduced phosphatase activity (Marklein and Houlton, 2012). To conclude, the interaction between bacterial diversity and enzyme activity was determined by nitrogen fertilizer application and affected by factors including soil physicochemical properties, soil types, climate, and farming patterns. The specific mechanism requires further investigation.

A majority of species of Acidobacteria are acidophilic. Redundancy analysis results showed that the relative abundance of Acidobacteria in surface soil was notably negatively correlated to the soil pH, which was confirmed by a previous study (Jones et al., 2009; Chu et al., 2010; Griffiths et al., 2011). The research of Wang H. et al. (2020) proved a significant positive connection between Acidobacteria and SOM, AP, and AN. Zhang et al. (2014) concluded that some subspecies of Acidobacteria were positively related to the activity of several key soil enzymes. In this paper, Acidobacteria in surface soil was found positively associated with AN, TN, AP, SOM, Alkaline phosphatase, and Sucrase. It was speculated that the positive relationship was originated from straw incorporation of previous crops prior to rice transplanting. Acidobacteria degraded straw as nutrients of soil, and the application of milk vetch in the organic farming pattern intensified the degradation activity of Acidobacteria. In subsurface soil, Proteobacteria was positively related to AN, TN, and SOM, which might be ascribed to the participation of nitrogen-fixing bacteria of Proteobacteria in the biochemical cycling of various nutrients in soil such as mineral carbon and nitrogen. Organic matter mineralization and degradation adds to the nitrogen source and thereby increases the relative abundance of nitrogen-fixing microorganisms in Proteobacteria (Chaudhry et al., 2012). However, Proteobacteria in surface soil was negatively related to most nutrients in this study. It was conjectured that some species of Proteobacteria were either adverse or unrelated to nutrient promotion. Further studies should focus more on specific bacterial communities of Proteobacteria since this phylum had the largest relative abundance in paddy soil. What’s more, this experiment also indicated that Chloroflexi was not relevant to most environmental factors despite its huge influence on bacterial community composition. It was probably due to high stability of Chloroflexi in paddy soil. Some species of Chloroflexi are well known because of their unique 3-HP structure, which is capable of fixing CO_2_ (Shih et al., 2017). Nevertheless, the genotype and phenotype of Chloroflexi vary greatly, and research on Chloroflexi strains is warranted to make up for the lack of knowledge of Chloroflexi.

## 5. Conclusion

Soil nutrients and enzyme activity decrease with the increasing soil depth. Organic farming can effectively improve soil nutrients and reduce nutrient loss, and has the potentiality to neutralize the alkaline soil in Gaoyou of Jiangsu Province. However, the organic farming mode will reduce the SOM content and urease activity in subsurface soil. Increased application of nitrogen fertilizer directly induces rising AN content and lowered pH values, while nitrogen reduction can activate a variety of enzymes. Besides, the overuse of nitrogen fertilizer will damage the diversity of soil microorganisms, while a proper reduction of nitrogen can improve the utilization rate of nitrogen fertilizer without affecting soil microbial diversity. The correlation analysis reveals a significant relationship of AN with diversity indices and enzyme activity. Organic farming upgrades markedly the community structure. Low nitrogen treatment under the organic farming mode can increase the relative abundance of Acidobacteria in surface soil and Nitrosomonadaceae in subsurface soil, thereby promoting the degradation of plant residues and boosting nitrification. Proteobacteria, Acidobacteria and Chloroflexi were the top 3 dominant phyla in paddy soil, and their total relative richness maintains stable. Proteobacteria and Acidobacteria play a critical part in the generation of soil nutrients. Therefore, we recommend an organic farming system with reduced application of nitrogen fertilizer for restoring fertility levels of soil in Gaoyou, Jiangsu Province.

## Data availability statement

The datasets presented in this study can be found in online repositories. The names of the repository/repositories and accession number(s) can be found at: https://www.ncbi.nlm.nih.gov/sra/PRJNA890158.

## Author contributions

CX: validation, formal analysis, investigation, data curation, and writing – original draft. YC: validation, investigation, and data curation. QZ: investigation. YL: methodology and investigation. JZ: investigation. XL: investigation. MJ: supervision. HZ: resources and project administration. LH: term, conceptualization, methodology, resources, and writing – review and editing. All authors contributed to the article and approved the submitted version.

## Funding

This study was financially supported by the National Key R&D Program of China Under Grant, projects number 2017YFD0300102. Modern Agricultural Development Projects of Jiangsu Province, projects number 2019-SJ-039-08-11.

## Conflict of interest

The authors declare that the research was conducted in the absence of any commercial or financial relationships that could be construed as a potential conflict of interest.

## Publisher’s note

All claims expressed in this article are solely those of the authors and do not necessarily represent those of their affiliated organizations, or those of the publisher, the editors and the reviewers. Any product that may be evaluated in this article, or claim that may be made by its manufacturer, is not guaranteed or endorsed by the publisher.
