# Supplementary material for: The effects of cultivation patterns and nitrogen levels on fertility and bacterial community characteristics of surface and subsurface soil
Source: Front Microbiol. 2023 Feb 16;14:1072228. doi: 10.3389/fmicb.2023.1072228 (PMC9978222; doi:10.3389/fmicb.2023.1072228)
Supplement: Supplementary file 1 [file Data_Sheet_1.PDF]

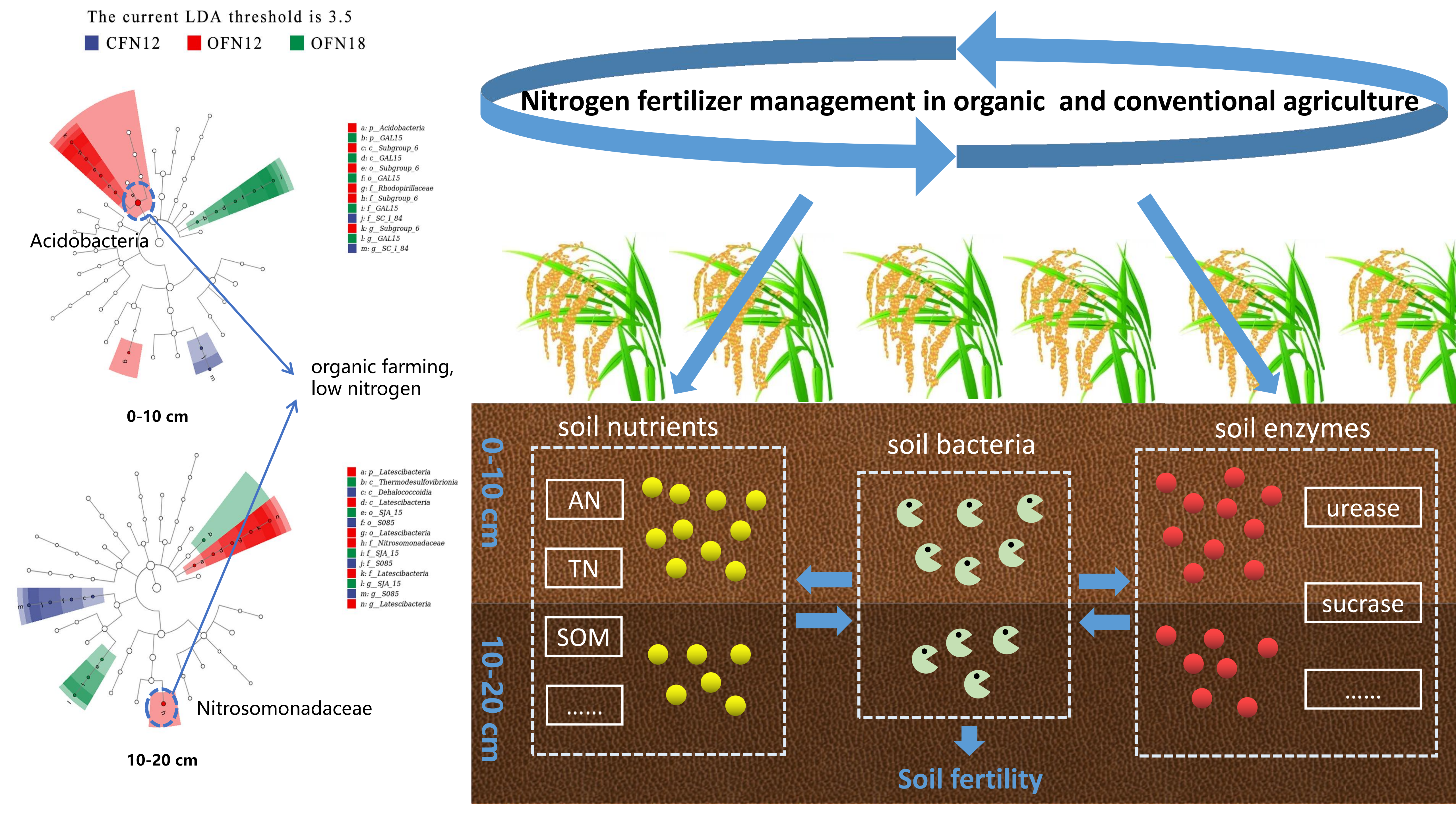

The current LDA threshold is 3.5
CFN12
OFN12
OFN18
a: p__Acidobacteria
b: p__GAL15
c: c__Subgroup_6
d: c__GAL15
e: o__Subgroup_6
f: o__GAL15
g: f__Rhodopirillaceae
h: f__Subgroup_6
i: f__GAL15
j: f__SC_I_84
k: g__Subgroup_6
l: g__GAL15
m: g__SC_I_84
Acidobacteria
0-10 cm
organic farming,
low nitrogen
a: p__Latescibacteria
b: c__Thermodesulfovibrionia
c: c__Dehalococcoidia
d: c__Latescibacteria
e: o__SJA_15
f: o__S085
g: o__Latescibacteria
h: f__Nitrosomonadaceae
i: f__SJA_15
j: f__S085
k: f__Latescibacteria
l: g__SJA_15
m: g__S085
n: g__Latescibacteria
Nitrosomonadaceae
10-20 cm
Nitrogen fertilizer management in organic and conventional agriculture
soil nutrients
AN
TN
SOM
……
soil bacteria
soil enzymes
urease
sucrase
……
0-10 cm
10-20 cm
Soil fertility
